# Screening for antioxidant and antibacterial activities of phenolics from *Golden Delicious* apple pomace

**DOI:** 10.1186/s13065-016-0195-7

**Published:** 2016-08-02

**Authors:** Tingjing Zhang, Xinyuan Wei, Zhuang Miao, Hamada Hassan, Yunbo Song, Mingtao Fan

**Affiliations:** 1 College of Food Science and Engineering, Northwest A&F University, Yang Ling, 712100 Shaanxi China; 2 Food Science Department, Faculty of Agriculture, Zagazig University, Zagazig, Egypt

**Keywords:** Polyphenols, Antioxidant activity, Antibacterial activity, Phloridzin, Phloretin

## Abstract

**Background:**

Synthetic antioxidants and antimicrobials are losing ground to their natural counterparts and therefore, the food industry has motivated to seek other natural alternatives. Apple pomace, a by-product in the processing of apples, is rich in polyphenols, and plant polyphenols have been used as food additives owing to their strong antioxidant and antimicrobial properties. The goal of this study was to screen the individual polyphenols with antioxidant and antimicrobial activities from the extracts (methanol, ethanol, acetone, ethyl acetate, and chloroform) of *Golden Delicious* pomace.

**Results:**

First, the polyphenolic compounds (total phenol content, TPC; total flavonoids, TFD; total flavanols, TFL) and antioxidant activities (AAs) with four assays (ferric reducing antioxidant power, FRAP; 1,1-diphenyl-2-picryhydrazyl radical scavenging capacity assay, DRSC; hydroxyl radical averting capacity assay, HORAC; oxygen radical absorbance capacity assay, ORAC) were analyzed. The results showed a significant positive correlation (*P* < 0.05) between AAs and TFD. Ethyl acetate extract (EAE) exhibited the highest TFD with a concentration of 1.85 mg RE/g powder (expressed as rutin equivalents), and the highest AAs (expressed as butylated hydroxytoluene (BHT) equivalents) with 2.07 mg BHT/g powder for FRAP, 3.05 mg BHT/g powder for DRSC, 5.42 mg BHT/g powder for HORAC, and 8.89 mg BHT/g powder for ORAC. Composition and AA assays of individual polyphenols from the EAE were then performed. Phloridzin and phloretin accounted for 46.70 and 41.94 % of TFD, respectively. Phloretin displayed the highest AA, followed by phloridzin. Finally, the antimicrobial activities of the EAE, phloridzin, and phloretin were evaluated. EAE displayed good inhibitory activities against *Staphylococcus aureus* with a minimum inhibition concentration (MIC) of 1.25 mg/ml and against *Escherichia coli* with a MIC of 2.50 mg/ml. Phloridzin and phloretin showed better inhibitory activities than the EAE, which were MICs of 0.50 and 0.10 mg/ml, respectively, against *S. aureus* and MICs of 1.50 and 0.75 mg/ml, respectively, against *E. coli*.

**Conclusions:**

Ethyl acetate was the best solvent of choice to extract natural products to obtain the maximum antioxidant and antibacterial benefits. Phloridzin and phloretin have the potential to be used as natural alternatives to synthetic antioxidants and antimicrobials.

## Background

*Golden Delicious* is one of the most popular cultivars (*Malus* × *Domestica*) in China due to its high yield, excellent quality, and good taste. The mean annual yield of *Golden Delicious* reaches 100,000 T in Lingyuan City, China, alone [1, 2]. However, *Golden Delicious* has its disadvantages with storage difficulties owing to its thin skin and its tendency for dehydration compared with other apple cultivars [3, 4]. In addition, respiration is prone to cause rapid fruit senescence and decline in quality during storage [2, 5]. Fruit rots such as ring rot, anthracnose and brown rot that happen often during the growth and storage period of these apples are another serious issue, especially apple ring rot [6]. All these drawbacks significantly shorten the storage life of *Golden Delicious*. Therefore, most *Golden Delicious* fruits are processed into cider, juice, jams, canned goods, or other products. During the apple processing procedures, a large quantity of apple pomace is generated; it contains peel, core, seed, calyx, stem, and soft tissue and accounts for 30 % of the weight of the original fruits [4, 7, 8].

In general, apple pomace contains over 60 different phenolic compounds [7–9]. Chemical studies on *Golden Delicious* pomace have revealed the presence of rutin, catechin, epicatechin, phloridzin, phloretin, chlorogenic acid, and quercetin glycosides [4, 7, 10]. These polyphenols are strong antioxidants that are able to counterbalance the free radicals which can cause human diseases such as cancers, heart diseases, diabetes, cardiovascular disease, Alzheimer’s disease, age-related functional decline, chronic diseases, and coronary heart diseases [11–14]. Moreover, these polyphenols with high redox potential are the most advantageous natural food additives that play a role in protection against oxidative damage or that act as reducing agents for protecting food from being damaged by unstable molecules such as reactive oxygen species [7, 12, 15, 16]. Additionally, plant polyphenols have been widely used because of their strong antiviral and antibacterial properties against foodborne pathogens, and therefore, could be applied as novel preservatives in the food industry [17–19]. However, apple pomace has been traditionally used as animal/fish feed or directly treated as an agricultural waste material without further processing, practices which not only cause serious environmental pollution but which waste resources as well.

Currently, synthetic antioxidants such as butylated hydroxytoluene (BHT) are the most commonly used antioxidants to preserve and maintain the freshness, nutritive value, flavour or colour of food products [20, 21]. However, the synthetic antioxidant BHT has been suspected of causing liver damage [22]. Chlorine, in the form of sodium hypochlorite at a certain concentration, is commonly used to disinfect products [22, 23], but it has limited efficacy and may be able to generate toxic chlorination by-products on food sources. Furthermore, the number of bacteria resistant to current synthetic antimicrobials has increased dramatically [22, 24, 25]. Thus, there is a great need for discovering new antioxidants and antimicrobials. Additionally, the mistrust of antioxidants or antimicrobials of synthetic origin due to their potential toxicity and carcinogenicity [22, 26, 27] has intensified the efforts for discovering other natural alternatives that are safer, more effective and environmentally friendly sanitation agents. Furthermore, phenolic compounds in apple pomace are an essential part of the human diet and are of noticeable interest due to their antioxidant and antibacterial properties [10].

The subject of this study was to screen *Golden Delicious* apple pomace for the phenolic compounds with antioxidant and antimicrobial activities that can partly or entirely replace the synthetic antioxidant BHT and the synthetic disinfector sodium hypochlorite. First, the total polyphenols and antioxidant activities of extracts obtained with five different organic solvents (methanol, ethanol, acetone, ethyl acetate, and chloroform) were evaluated. The major individual polyphenols in the extract that exhibited the highest antioxidant activity were then analyzed by high performance liquid chromatography coupled with a diode array detector (HPLC–DAD), and the antibacterial activity of this extract was determined by the agar disk diffusion method. Finally, the natural extract was compared with the synthetic antioxidant BHT and the synthetic disinfector sodium hypochlorite to assess its potential as an alternative natural antioxidant and antimicrobial.

## Methods

### Plant materials, chemicals and reagents

*Golden Delicious* ripe fruits were collected in the experimental orchard of the Horticultural Institute of Northwest A&F University (Yangling, Shaanxi, China). Apple pomace was isolated using a fruit squeezer (MY-610, SKG, Guangdong, China), frozen in liquid N_2_ immediately after harvest, and then stored at −80 °C. Folin-Ciocalteu reagents, AlCl_3_, industrial grade antioxidants BHT with a purity of 99 %, and 1,1-diphenyl-2-picrylhydrazyl (DPPH) were purchased from Sigma-Aldrich (St. Louis, MO, USA). Individual phenol standards with purities >98 % were purchased from Chengdu Must Bio-Technology Co., Ltd. (Chengdu, China). All other chemicals and reagents were of analytical grade.

### Extraction of phenolic compounds

The extraction of polyphenols was performed according to the method described by Ran et al. with minor modifications [28]. Flesh apple pomace was ground into powder in liquid N_2_. Then, 100 g of powder was extracted with 500 ml of methanol, ethanol, acetone, ethyl acetate, and chloroform, respectively, in an ultrasonic bath at 37 °C for 40 min. All the produced extracts were dried under negative pressure in rotary evaporation at 40 °C and then re-dissolved in 10 ml of edible alcohol. The five extracts were filtered through a 0.45-μm membrane (Millipore) and stored in a refrigerator at 4 °C until analysis.

### Phenolic compounds analysis

The total polyphenol content (TPC) of five extracts was determined by a Folin-Ciocalteu method [28] and calculated as milligram gallic acid equivalent per gram of powder (mg GAE/g powder).

Total flavonoids (TFD) were determined according to the method based on the formation of flavonoid complex with aluminium [22] and expressed as milligram rutin equivalent per gram of powder (mg RE/g powder).

Total flavanols (TFL) were determined according to the method of Leyva-Corral et al. [9] and calculated as milligram epicatechin equivalent per gram of powder (mg EE/g powder). The calculation formula is provided as: *X* = (*A* × *m*_0_)/(*A*_0_/*m*), where *X* is the total flavanol content of the extracts; *A* is the absorbance of the extracts at 640 nm; *A*_0_ is the absorbance of epicatechin (1.00 mg/ml) at 640 nm; *m*_0_ is the content of epicatechin (100 μg); and *m* is the wet weight of apple pomace (1.00 g).

### Antioxidant activity assays

#### Ferric reducing antioxidant power (FRAP) assay

The FRAP assay was carried out according to the method described by Khaled-Khodja et al. [22]. Serially diluted BHT solutions (0, 0.15625, 0.3125, 0.625, 1.25, 2.50, and 5.00 mg/ml) were used to plot the standard curve.

#### DPPH radical scavenging capacity (DRSC) assay

The DRSC assay was conducted according to the previous method [9]. BHT solution (0–5.00 mg/ml) was used to plot the standard curve. The DRSC was calculated according to the equation: DRSC (100 %) = [1 − (A_sample_/A_control_)] × 100 %.

#### Hydroxyl radical averting capacity (HORAC) assay

The HORAC assay was performed as developed by Denev et al. [13] that measured the metal-chelating activity of extracts in the conditions of Fenton-like reactions employing a Co(II) complex and, hence, determined the ability of the extracts to protect against the formation of hydroxyl radicals. The protective effects of the extracts and BHT were measured by assessing the area under the fluorescence decay curve (AUC) relative to that of the control. BHT solutions (0, 0.15625, 0.3125, 0.625, 1.25, 2.50, and 5.00 mg/ml) were used to plot the standard curve.

#### Oxygen radical absorbance capacity (ORAC) assay

The ORAC assay was performed according to the method of Denev et al. [13] that measured the antioxidant scavenging activity against peroxyl radical generated by the thermal decomposition of 2,2′-azobis [2-methylpropionamidine] dihydrochloride (AAPH) at 37 °C. Fluorescein (FL) was used as the fluorescent probe. Loss of FL fluorescence was an indication of the extent of damage from its reaction with peroxyl radicals. The antioxidant scavenging activity of extracts against peroxyl radicals was evaluated by assessing the AUC. Ethanol was used instead of samples as the control in the four antioxidant activity assays, and the results were expressed as milligram BHT equivalents per gram of powder (mg BHT/g powder).

### Identification and quantification of individual polyphenols

HPLC–DAD was used to identify and quantify individual polyphenols in the extract according to retention time and the standard curve regression equations of the standards [7]. The HPLC–DAD (Shimadzu, Kyoto, Japan) detection was performed with a WondaSil® C_18_ column (4.6 × 250 mm, ID = 5 µm) by a binary programme with solvent systems including water (0.01 % phosphoric acid) as Solvent A and methanol (100 %) as Solvent B. The programme was described as follows: 0–20 min, 20–50 % B; 20–25 min, 50–70 % B; 25–30 min, 70–80 % B; 30–35 min, 80–20 % B; 35–45 min, 20 % B. The solvent flow rate was 0.7 ml/min. The UV detector was set to the wavelength of 280 nm, and the injection volume was 10 µl.

### Antibacterial activity

The in vitro antibacterial activities of samples were tested against Gram-positive bacteria (*Staphylococcus aureus* ATCC6538) and Gram-negative bacteria (*Escherichia coli* ATC10536) using the agar diffusion method. The activities were evaluated by measuring the diameter of inhibition zone (DIZ) in millimetres and the MIC according to the method described by Barreca et al. [17]. Ethanol was used as the negative control, and sodium hypochlorite solution (0.20 mg/ml, SHS) was used as the positive control under the same conditions.

### Statistical analysis

All data are expressed as the mean ± SD of triplicate measurements. The statistically significant differences among mean values at the level of significance (P < 0.05) were evaluated with the paired *t* test in SPSS (version 19.0).

## Results and discussion

### Polyphenolic compounds analysis

Polyphenolic compounds, in particular flavonoids, have been suggested to be the major contributors to the antioxidant capacity of plant extracts [4, 7, 9, 10, 15, 22]. Some diverse biological activities, such as antimicrobial activity, are also thought to be related to polyphenolic compounds [17, 19, 27, 29]. To validate this notion, the TPC, TFD, and TFL of five extracts from *Golden Delicious* pomace were evaluated. As shown in Table 1, the TPC of the five extracts varied significantly (*P* < 0.05) according to the extraction medium, ranging from 1.62 to 3.05 mg GAE/g powder. The highest level of TPC was detected in the methanol extract (ME), whereas the lowest was observed in the chloroform extract (CE). Lou et al. and Massias A et al. revealed that the yields of phenols depended on the type of the extraction medium, and methanol was an ideal extractant for the separation of phenolics [7, 30]. In this study, the TPC of the ME from *Golden Delicious* pomace was also in accordance with a previous report by Junjian et al. who showed the TPC of a ME from apple pomace was 2.98 mg GAE/g powder [28]. In other cases, extracts of apple pomace exhibited lower TPC, with 0.64 mg GAE/g powder, 1.48 mg GAE/g powder, and 1.96 mg GAE/g powder, respectively [9, 31, 32], whereas the previous report by Massias A et al. showed the TPC of a methanolic extract from apple pomace was 7.92 mg GAE/g powder [7]. These differences could be attributable to biological factors (genotype, organ and apple cultivars), as well as edaphic and environmental (temperature, salinity, waterstress and light intensity) conditions. Moreover, the solubility of phenolic compounds is governed by the type of solvent used, the degree of polymerization of phenolics, and their interaction.

**Table 1:** Polyphenolic compounds of extracts

Extracts	TPC	TFD	TFL
Methanol	3.05 ± 0.82^a^	1.53 ± 0.17^b^	1.13 ± 0.11^a^
Ethanol	2.87 ± 0.75^a^	1.57 ± 0.14^b^	1.08 ± 0.12^a^
Acetone	2.15 ± 0.35^c^	0.99 ± 0.10^c^	0.81 ± 0.11^b^
Ethyl acetate	2.51 ± 0.42^b^	1.85 ± 0.13^a^	0.54 ± 0.10^c^
Chloroform	1.62 ± 0.23^d^	0.82 ± 0.10^c^	0.57 ± 0.10^c^

All values are expressed as the mean ± standard deviation (n = 3)

*TPC* total phenolic compounds (mg GAE/g powder), *TFD* total flavonoids (mg RE/g powder), *TFL* total flavanols (mg EE/g powder)

^a–d^Column wise values with different superscripts of this type indicate significant differences (P < 0.05)

The TFD of the extracts ranged from 0.82 to 1.85 mg RE/g powder among the five organic solvents. The EAE showed the maximum quantity of TFD and the lowest amount was also observed in the chloroform extract. Previous studies have shown that apple is rich in flavonoids, especially abundant in the apple peel and seeds [4, 7–10, 15, 17, 28, 31, 32]. Cao et al. separated six polyphenolic compounds including four quercetin glycosides, phloridzin and phloretin in the ethyl acetate extract of apple pomace [8]. Quercetin glycosides are flavonols, and both phloridzin and phloretin are categorized as the dihydrochlcones, but these two categories are the subclasses of flavonoids [33]. Kołodziejczyk et al. isolated four types of flavonoids (quercetin, kaempferol, naringenin, and phloridzin) in the chloroform extract of plants [34]. In terms of TFL, it showed a strong-link behavior in contrast to TPC in the five extracts.

Both methanol and ethanol are strong polar solvents that are efficient in degrading cell walls and releasing polyphenols from cells [3]. Additionally, the polarity and solvency of methanol and ethanol were extremely similar. Therefore, these two extraction medium showed insignificant differences and exhibited the highest levels of TPC and TFL (Table 1); these results are in good accordance with the principle that dissolution of polyphenols would be similar in solvents with similar material structures. Interestingly, the best extraction performance for total flavonoids (TFD) from apple pomace was achieved with the extraction medium of ethyl acetate (Table 1), whose polarity was weaker than that of methanol and ethanol. In previous studies, the best preparation of flavonoids from *Malus domestica*, *Launaea procumbens*, *kumquat*, and *Spanish olive* cultivars was obtained with the use of ethyl acetate [8, 30, 35, 36]. It has been reported that ethyl acetate is the optimal reagent for isolation of active substances from plant materials [37, 38]. In present study, it was confirmed that among all the employed organic solvent mixtures, ethyl acetate was the most effective solvent for the preparation of flavonoid-rich extracts.

### Antioxidant activity analysis

Numerous studies have demonstrated that apple polyphenols are effective scavengers of physiologically relevant reactive oxygen and nitrogen species in vitro [4, 7, 9, 31]. Moreover, the radical-scavenging and antioxidant properties of apple polyphenols are frequently cited as important contributors in different models of human chronic diseases [12–14]. Table 2 presents the antioxidant activities (AAs) of the five extracts as determined in the following four assays: FRAP, DRSC, HORAC, and ORAC assays. The four AA assays (FRAP, DRSC, HORAC, ORAC) varied significantly (P < 0.05) according to the extraction medium and displayed the same trend that paralleled the evolution of TFD in five extracts, suggesting that flavonoids were the major active component in these extracts. Accordingly, the EAE exhibited the highest AA, followed by the methanol extract and ethanol extract, and the lowest AA was found in the chloroform extract. The AA values obtained with the four methods varied significantly (P < 0.05) within the same extraction mediums, revealing a ranking order as follows: ORAC > HORAC > DRSC > FRAP. The reasons for these variations might be attributed to the interference effect of the extraction medium and non-antioxidant constituents.

**Table 2:** Antioxidant capacity of extracts

Extracts	FRAP	DRSC	HORAC	ORAC
Methanol	1.42 ± 0.14^bD^	2.13 ± 0.13^bC^	3.46 ± 0.19^bB^	5.41 ± 0.24^bA^
Ethanol	1.36 ± 0.12^bD^	2.11 ± 0.10^bC^	3.50 ± 0.23^bB^	5.73 ± 0.61^bA^
Acetone	1.07 ± 0.07^cD^	1.19 ± 0.11^bcC^	2.05 ± 0.18^cB^	2.70 ± 0.28^cA^
Ethyl acetate	2.07 ± 0.16^aD^	3.05 ± 0.14^aC^	5.42 ± 0.19^aB^	8.89 ± 0.62^aA^
Chloroform	0.70 ± 0.09^dD^	1.09 ± 0.08^cC^	1.52 ± 0.13^dB^	1.99 ± 0.13^cA^

All values are expressed as the mean ± standard deviation (n = 3)

*FRAP* ferric reducing power expressed as milligram BHT equivalents per gram of powder (mg BHT/g powder), *DRSC* DPPH radical scavenging capacity expressed as milligram BHT equivalents per gram of powder (mg BHT/g powder), *HORAC* hydroxyl radical averting capacity expressed as milligram BHT equivalents per gram of powder (mg BHT/g powder), *ORAC* oxygen radical absorbance capacity expressed as milligram BHT equivalents per gram of powder (mg BHT/g powder)

^a–d^ Column wise values with different superscripts of this type denote significant differences (P < 0.05)

^A–D^ Line wise values with different superscripts of this type denote significant differences (P < 0.05)

In general, extracts with high flavonoid content possess excellent antioxidant activity [22, 29, 31, 32]. Moreover, flavonoids in plant extracts have been considered the main bioactive compounds with antioxidant activity [7, 9, 35]. Furthermore, both antioxidant activity and total flavonoid contents of the extracts in the present study showed the same order. Thus, correlation coefficient (r) was calculated to estimate the correlation between TFD and the AAs (FRAP, DRSC, HORAC, ORAC) of the EAE (Table 3). The AA determined by ORAC had significant positive correlations (P < 0.05) with TFD, whereas the other three AA measurement methods were highly correlated (P < 0.01) with TFD, confirming that total flavonoid content was the main contributor to the antioxidant activities and could be used as an indicator for predicting AAs of plant extracts. In addition, four parameters (FRAP, DRSC, HORAC, ORAC) of AAs were highly correlated (P < 0.01) with each other. These results were in agreement with those reported in previously published studies [22, 30, 38]. Our results might be explained by the use of the same mechanisms or by the same polyphenols being active as antioxidants in the four assays.

**Table 3:** Correlation matrix between total flavonoids and antioxidant activities of EAE

Assays	Correlation coefficient (r)			
	TFD	FRAP	DRSC	HORAC
FRAP	0.997**	1	0.996**	0.983**
DRSC	0.990**	0.996**	1	0.993**
HORAC	0.975**	0.983**	0.993**	1
ORAC	0.952*	0.970**	0.971**	0.984**

*FRAP* ferric reducing power, *DRSC* DPPH radical scavenging capacity, *HORAC* hydroxyl radical averting capacity, *ORAC* oxygen radical absorbance capacity

* Significant correlation (P < 0.05)

** Highly significant correlation (P < 0.01)

### Identification, quantification and AA evaluation of individual polyphenols in EAE

Flavonoids, which act as powerful inhibitors of food oxidation due to their strong antioxidant activities, make up an ubiquitous class of secondary metabolites that are mainly derived from human foods such as fruit, vegetables, nuts, seeds, stems, flowers, tea, wine, olive oil, orange, propolis, and honey [4, 11, 36, 39]. To screen the main polyphenols that are responsible for the antioxidant properties of the EAE, individual polyphenols were identified and quantified by comparisons with available standards based on recorded retention time (Table 4). Major individual polyphenols in the EAE included gallic acid, chlorogenic acid, procyanidin B_2_, quercetin-3-*O*-rthamnoside, syringing, hyperin, phloretin, querecetin-3-*O*-pentoside, phloridzin and quercetin, which are the typical polyphenols in apples [7, 8]. The content of these individual polyphenols varied significantly (*P* < 0.05). As shown in Table 4, one dihydrochalcone, identified as phloridzin, was measured to be the most abundant polyphenol (0.86 mg/g powder). Another dihydrochalcone, identified as phloretin, was the second abundant polyphenol (0.78 mg/g powder). Phloridzin has been reported to be the predominant phenolic compound and represents more than 90 % of the soluble phenolics in apple pomace [17]. Phloretin is the flavone aglycone of phloridzin and can be converted into phloridzin in the presence of phloretin-2′-*O*-glycosyltransferase and activated uridine diphosphate glucose [39]. Both of these two dihydrochalcones belong to the same chemical class of flavonoids and are characterized structurally by two phenolic rings connected through a flexible open-chain three-carbon linker [33]. Both of them exhibited a wide spectrum of interesting and pharmacological bioactivities [33, 39]. Antioxidant activity has reported to be the most prominent bioactivity [33]. In this study, the total content of phloridzin and phloretin accounted for 65.18 % of TPC and 88.64 % of TPD in the EAE, respectively. This leads us to speculate that phloridzin and phloretin were the main components responsible for the antioxidant activity of the EAE. However, other components of the EAE, such as procyanidin B_2_, hyperin, quercetin-3-*O*-pentoside, and quercetion-3-*O*-rhamnoside, were reported to possess higher antioxidant activity than either phloridzin or phloretin at the same concentration [7, 31]. To determine the major contributors to the antioxidant activity of the EAE, the AAs of reference standards (procyanidin B_2_, phloridzin, hyperin, quercetin-3-*O*-pentoside, quercetion-3-*O*-rhamnoside, and phloretin) with the same concentrations as that were observed in the EAE were determined (Table 5) as well. The results indicated that 7.75 mg/ml of the reference standard phloretin displayed the highest AA, followed by 8.63 mg/ml of the reference standard phloridzin, and both of them were responsible for the antioxidant activity of the EAE up to 50 %. Notably, phloretin showed higher antioxidant activity than phloridzin, even though the content of phloretin tested in this study was lower than that of phloridzin. Similar results were also observed in other reports [7, 10, 31, 40]. Additionally, both phloridzin and phloretin displayed the highest scavenging activity against peroxyl radicals among the four AA assays; this result might be explained by the fact that the peroxide anion was capable of destroying the structure of the flavonoids. Phloridzin and phloretin, hence, have the potential to be isolated from EAE as natural antioxidants for used in the food industry, especially for removing the peroxyl radicals formed in food.

**Table 4:** Identification and quantification of individual phenols in ethyl acetate extract

Peaks	RT	Individual phenols	Standard equations/[*Y* = ax + b]	R^2^	Contents (mg/g powder)
1	3.95	Gallic acid	*Y* = 0. 208 *x* + 0.009	0.9987	0.06^f^ ± 0.01
7	24.64	Chlorogenic acid	*Y* = 0.108 *x* + 0.006	0.9981	0.06^f^ ± 0.01
8	25.97	Procyanidin B_2_	*Y* = 0.541 *x* + 0.097	0.9993	0.14^c^ ± 0.02
11	28.82	Phloridzin	*Y* = 0.935 *x* + 0.470	0.9992	0.86^a^ ± 0.25
12	29.64	Syringin	*Y* = 0.293 *x* + 0.017	0.9943	0.07^ef^ ± 0.02
13	30.26	Hyperin	*Y* = 0.489 *x* + 0.038	0.9983	0.11^d^ ± 0.08
14	30.66	Quercetin-3-*O*- pentoside	*Y* = 0.453 *x* + 0.036	0.9961	0.11^d^ ± 0.07
15	31.13	Quercetin-3-rhamnoside	*Y* = 0.503 *x* + 0.056	0.9957	0.11^d^ ± 0.07
18	32.90	Phloretin	*Y* = 0.830 *x* + 0.215	0.9995	0.78^b^ ± 0.23
20	35.37	Quercetin	*Y* = 0.301*x* + 0.028	0.9975	0.08^e^ ± 0.01

Values are expressed as the mean ± standard deviation (n = 3)

*Y* is the relative absorption area of corresponding reference standard at 280 nm. *x* is the content of corresponding reference standard

*RT* retention time (min), *R* ^*2*^ determination coefficient

^a–f^Column wise values with different superscripts of this type denote significant differences (P < 0.05)

**Table 5:** Antioxidant activities of six individual phenol standards

Individual phenols	FRAP	DRSC	HORAC	ORAC	Concentration (mg/ml)
Procyanidin B_2_	0.10 ± 0.01^c^	0.25 ± 0.03^c^	0.79 ± 0.05^c^	1.23 ± 0.05^c^	1.38 ± 0.02
Phloridzin	0.62 ± 0.05^bD^	0.89 ± 0.05^bC^	1.16 ± 0.08^bB^	2.01 ± 0.07^abA^	8.63 ± 0.05
Hyperin	0.08 ± 0.01^d^	0.15 ± 0.02^d^	0.37 ± 0.02^d^	0.78 ± 0.02^d^	1.07 ± 0.01
Quercetin-3-pentoside	0.06 ± 0.01^e^	0.13 ± 0.01^e^	0.32 ± 0.02^d^	0.75 ± 0.03^d^	1.06 ± 0.01
Quercetin-3-rhamnoside	0.07 ± 0.01^de^	0.14 ± 0.01^de^	0.35 ± 0.01^d^	0.73 ± 0.04^d^	1.14 ± 0.02
Phloretin	1.88 ± 0.07^aB^	1.27 ± 0.08^aC^	1.86 ± 0.06^aB^	2.58 ± 0.10^aA^	7.75 ± 0.04

Values are expressed as the mean ± standard deviation (n = 3)

*FRAP* ferric reducing power expressed as milligram BHT equivalents per milliliter of ethyl acetate extract (mg BHT/ml ethyl acetate extract), *DRSC* DPPH radical scavenging capacity expressed as milligram BHT equivalents per milliliter of ethyl acetate extract (mg BHT/ml ethyl acetate extract), *HORAC* hydroxyl radical averting capacity expressed as milligram BHT equivalents per milliliter of ethyl acetate extract (mg BHT/ml ethyl acetate extract), *ORAC* oxygen radical absorbance capacity expressed as milligram BHT equivalents per milliliter of ethyl acetate extract (mg BHT/ml ethyl acetate extract)

^a–e^Column wise values with different superscripts of this type denote significant differences (P < 0.05)

^A–D^Line wise values with different superscripts of this type denote significant differences (P < 0.05)

### Antibacterial activity analysis

The use of natural compounds as antibacterial agents has been highlighted to be an alternative to synthetic antioxidant compounds due to their reduced side effects, low cost of drug development, and lower likelihood of stimulating multiple drug resistance [17]. In this context, the antimicrobial activities of phloridzin and phloretin as well as EAE were analyzed against Gram positive and negative bacterial strains. The DIZs and MICs obtained are listed in Table 6. All samples were observed to be active against both *S. aureus* and *E. coli* with zones of inhibition between 16.09 and 39.17 mm for *S. aureus* and between 12.57 and 28.25 mm for *E. coli* at the tested concentrations. The phloretin standard, with a concentration of 5.00 mg/ml, had a maximum inhibition zone against *S. aureus* and *E. coli*, whereas the EAE, with a concentration of 5.00 mg GAE/ml, had the minimum inhibition zone against both *S. aureus* and *E. coli*. Khaled-Khodja et al. [22] and Barreca et al. [17] have reported similar findings. Except for DIZ, the MICs also varied significantly (P < 0.05) from phloridzin to sodium hypochlorite solution (SHS) for both *S. aureus* and *E. coli*. For *S. aureus*, phloretin and the positive control SHS displayed the strongest antibacterial activity, followed by phloridzin, and the EAE exhibited the weakest antibacterial activity. For *E. coli*, SHS displayed the strongest antibacterial activity, followed by phloretin and phloridzin. The EAE still showed the lowest activity. The relatively lower antibacterial activity of the EAE could be ascribed to the fact that the phloridzin and phloretin standards used in this study were of the chromatographic purity ≥98 %, however the natural phloridzin and phloretin extracted from plant materials were often conjugated with organic acids, polysaccharides, DNA, proteins, and other polyphenols [7, 8, 28] that could reduce the antibacterial activity.

**Table 6:** Antibacterial activity of EAE (inhibition zone and MIC)

Samples	DIZ (mm)		MIC (mg/ml)	
	*S. aureus*	*E. coli*	*S. aureus*	*E. coli*
Phloridzin	30.15 ± 1.66^b^	17.05 ± 1.04^c^	0.50 ± 0.05^b^	1.50 ± 0.12^c^
Phloretin	39.17 ± 2.71^a^	28.25 ± 1.67^a^	0.10 ± 0.02^a^	0.25 ± 0.10^b^
Ethyl acetate extract	16.09 ± 1.07^d^	12.57 ± 1.34^d^	1.25 ± 0.11^c^	2.50 ± 0.14^d^
SHS	21.33 ± 1.25^c^	23.75 ± 1.95^b^	0.10 ± 0.05^a^	0.15 ± 0.03^a^

Values are expressed as the mean ± standard deviation (n = 3)

The concentration of phloridzin and phloretin tested in DIZ was set to 5.00 mg/ml, and the concentration of ethyl acetate extract was set to 5.00 mg GAE/ml extract

*SHS* sodium hypochlorite solution with a content of 0.20 mg/ml, *DIZ* diameter of inhibition zone, *MIC* minimum inhibition concentration

^a–d^ Column wise values with different superscripts of this type denote significant differences (P < 0.05)

The results obtained through the determination of DIZs and MICs also suggested that (i) dihydrochalcone (phloretin and phloridzin) displayed a more effective antimicrobial impact against Gram-positive *S. aureus* than against Gram-negative *E. coli* and that (ii) the addition of glucose to the basic structure of dihydrochalcone determined a net reduction of antimicrobial activity. Previous studies reported the same findings that phloretin was particularly active against *S. aureus* [17, 41]. The *S. aureus* strain causes food poisoning by releasing enterotoxins into food, and toxic shock syndrome by release of super-antigens into the blood stream [22]. Therefore, preventing the growth and propagation of *S. aureus* has been focused on searching for new natural nontoxic compounds to inhibit its growth or to enhance adherence to basic inhibitors as an infection control practice. Combined with the antioxidant activities analysis, antibacterial activity tests revealed that phloretin and phloridzin are potential natural antioxidant and antibacterial agents that could be used to replace synthetic antioxidants and antiseptics, especially phloretin. However, problems that still need to be addressed include the weaker aqueous solubility, lower absorbability, poor purity, and instability of phloretin because these drawbacks could lead to the reduction in antioxidant and antibacterial activities as well.

## Conclusion

In this study, phenolic compounds were isolated from *Golden Delicious* pomace with five organic solvents (methanol, ethanol, acetone, ethyl acetate, and chloroform), and the antioxidant activities of these extracts were determined. The highest levels of TPC and TFL were found in the methanol extract. The ethyl acetate extract showed the highest amount of TFD, whereas the lowest amounts of TPC, TFD and TFL were found in the chloroform extract. Both the antioxidant activity and TFD of the extracts had the same order: ethyl acetate extract > methanol extract ≈ ethanol extract > acetone extract > chloroform extract. Additionally, the four antioxidant activity assays within the same extraction medium revealed the following order: ORAC > HORAC > DRSC > FRAP. Phloridzin and phloretin were measured to be the predominant components in the extract and displayed higher antioxidant activity than the ethyl acetate extract, therefore, these flavonoids are considered to be responsible for the antioxidant properties of the extract. In addition to antioxidant activity, phloretin, phloridzin, and ethyl acetate extract all have activities against both *S. aureus* and *E. coli*. Phloretin, which accounted for 41.94 % of TFD in ethyl acetate extract, has the highest antimicrobial activity against both *S. aureus* and *E. coli*, and in particular against *S. aureus* ATCC 6538. And, *S. aureus* was more sensitive to the ethyl acetate extract than *E. coli*. Notably, phloridzin showed a relatively higher antimicrobial activity and was able to take the place of phloretin due to its stronger water solubility, better stability and higher content in apple pomace. These experimental results provide the basis for the development of promising natural antimicrobial agents possessing antioxidant activity and for supporting the potential use of apple pomace extracts as food supplements or the potential applications of these natural antioxidants in the pharmaceutical and manufacturing industries.
